# Dose‐adjusted EPOCH‐R is not superior to sequential R‐CHOP/R‐ICE as a frontline treatment for newly diagnosed primary mediastinal B‐cell lymphoma: Results of a bi‐center retrospective study

**DOI:** 10.1002/cam4.4387

**Published:** 2021-11-24

**Authors:** Yael Morgenstern, Shlomzion Aumann, Neta Goldschmidt, Moshe E. Gatt, Boaz Nachmias, Netanel A. Horowitz

**Affiliations:** ^1^ Department of Hematology and Bone Marrow Transplantation Rambam Health Care Campus Haifa Israel; ^2^ Department of Hematology Hadassah Medical Center and Faculty of Medicine Hebrew University of Jerusalem Jerusalem Israel; ^3^ The Ruth and Bruce Rappaport Faculty of Medicine Technion – Israel Institute of Technology Haifa Israel

**Keywords:** DA‐EPOCH‐R, Primary mediastinal B‐cell lymphoma (PMBCL), radiation therapy (RT), R‐CHOP/R‐ICE

## Abstract

**Purpose:**

Primary mediastinal B‐cell lymphoma (PMBCL) is a rare subtype of diffuse large B‐cell lymphoma (DLBCL). Despite its aggressive course, PMBCL is considered curable. While in recent years dose‐adjusted (DA) EPOCH‐R (rituximab, etoposide, prednisone, vincristine, cyclophosphamide and doxorubicin) has become widely endorsed as first‐line therapy for newly‐diagnosed PMBCL, the optimal treatment for this disease and the role of radiotherapy (RT) remains unclear. DA‐EPOCH‐R provides good clinical outcomes, albeit is associated with short‐ and long‐term toxicity. To address this issue, the current retrospective bi‐icenter analysis compared efficacy and toxicity of DA‐EPOCH‐R and a less toxic R‐CHOP/R‐ICE regimen used for the treatment of newly‐diagnosed PMBCL.

**Patients and Methods:**

The study included all patients with a histologically confirmed PMBCL diagnosis treated with DA‐EPOCH‐R or R‐CHOP/R‐ICE between 01/2013‐12/2020 at two tertiary medical centers. Patient demographic and clinical data were derived from institutional electronic medical records. The analysis included 56 patients: 31 received DA‐EPOCH‐R and 25 – R‐CHOP/R‐ICE.

**Results:**

At a median follow‐up of 1.9 years (IQR 3.1 years), similar progression‐free survival (2.1 versus 2.4 years; *p* = 0.7667), overall survival (2.5 versus 2.7 years; *p* = 0.8047) and complete response (80%) were observed in both groups. However, DA‐EPOCH‐R was associated with significantly longer hospitalization required for its administration (*p* < 0.001) and a trend for higher frequency of infections, stomatitis, thrombotic complications and febrile neutropenia‐related hospitalizations.

**Conclusion:**

DA‐EPOCH‐R and R‐CHOP/R‐ICE provide similarly encouraging outcomes in newly‐diagnosed PMBCL patients. R‐CHOP/R‐ICE is associated with lower toxicity and significantly reduced hospitalization. Our findings suggest that this regimen may be considered as an alternative to DA‐EPOCH‐R in this patient population.

## Graphical abstract

Primary mediastinal B‐cell lymphoma (PMBCL) is a rare subtype of diffuse large B‐cell lymphoma that is considered curable despite its aggressive course. DA‐EPOCH‐R, widely employed as a first‐line therapy for newly diagnosed PMBCL, offers good clinical results, but its utility is hampered by high rates of short‐ and long‐term toxicity. Our findings suggest that the use of R‐CHOP/R‐ICE could be consider as an alternative to DA‐EPOCH‐R in PMBCL patients, since the former regimen, while providing similarly encouraging outcomes, is associated with lower toxicity and significantly reduced hospitalization.

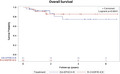

## 1 INTRODUCTION

Primary mediastinal B‐cell lymphoma (PMBCL), previously considered a subtype of diffuse large B‐cell lymphoma (DLBCL) is a rare aggressive hematological malignancy characterized by distinct clinical, morphological, and immunophenotypical features.^1^, ^2^, ^3^, ^4^, ^5^ Clinically, PMBCL usually affects women in the age of 25–40 years and typically presents with bulky mediastinal involvement. Morphologically and biologically, it is shown to be related to nodular sclerosing Hodgkin lymphoma.^1^ Formerly, the R‐CHOP regimen (rituximab, cyclophosphamide, doxorubicin, vincristine, and prednisolone) and subsequent consolidation radiotherapy (RT) had been commonly used as a first‐line treatment, resulting in a 5‐year progression‐free survival (PFS) of 80% and a 5‐year overall survival (OS) of 87%.^6^, ^7^, ^8^ However, several retrospective studies have reported a high primary induction failure rate (21%) with R‐CHOP, raising the possibility of limited efficacy of this regimen in PMBCL.^9^, ^10^ Comparable rates of PFS and OS have been demonstrated with MACOP‐B (methotrexate, doxorubicin, cyclophosphamide, vincristine, prednisone, and bleomycin) or VACOP‐B (etoposide, doxorubicin, cyclophosphamide, vincristine, prednisone, and bleomycin) followed by RT.^11^, ^12^ Yet, given the acute and long‐term RT‐associated toxicity, including cardiovascular involvement and secondary malignancies, especially in the relatively young female population, several strategies intending to mitigate unnecessary exposure to RT have been considered. Nevertheless, PMBCL is known to be a radiosensitive disease,^13^, ^14^, ^15^, ^16^ albeit several studies have demonstrated the main prognostic impact of RT in patients with residual disease, as evidenced in the end‐of‐treatment (EOT) positron emission tomography‐computed tomography (PET‐CT) result, while the outcomes of patients in complete remission (CR) have been excellent even in the no‐RT setting.^7^, ^11^, ^17^, ^18^, ^19^, ^20^, ^21^ Remarkably, using PET‐CT as a guide to decide regarding the necessity of RT in PMBCL patients treated with R‐CHOP, resulted in a 64% reduction in the number of patients who received consolidative RT, without affecting their outcomes.^6^, ^18^, ^22^, ^23^

In the last decade, dose‐adjusted etoposide, prednisone, vincristine, cyclophosphamide, doxorubicin, and rituximab (DA‐EPOCH‐R) has become widely accepted as a first‐line treatment for PMBCL, allowing to avert the need for RT. A single‐center prospective phase II study has reported a 93% event‐free survival (EFS) of and 97% OS of at a median follow‐up of 63 months following 6–8 cycles of DA‐EPOCH‐R with no need for subsequent RT in most patients.^16^, ^24^, ^25^ However, DA‐EPOCH is administered as a continuous infusion via a central venous catheter (CVC), mostly in the inpatient setting, and is associated with high toxicity, including thrombocytopenia and febrile neutropenia requiring hospitalization.^26^ All these factors may somewhat offset the benefits of this therapeutic approach. The dose‐dense R‐CHOP/R‐ICE (ifosfamide, cyclophosphamide, and etoposide) is an alternative outpatient regimen suggested for PMBCL management. It is limited to chemotherapy only, without need for RT. Treatment with this regimen does not require CVC insertion and is reported to provide 3‐year OS and PFS rates of 88% and 78%, respectively.^27^, ^28^, ^29^ However, data from prospective randomized trials are limited because of disease rarity.

The current retrospective study has compared the efficacy, including PFS and OS, as well as the toxicity profile of two intensive chemotherapy regimens including DA‐EPOCH‐R and the sequential R‐CHOP/R‐ICE regimen employed as frontline treatments for newly diagnosed PMBCL.

## 2 MATERIALS AND METHODS

### 2.1 Study design and patient population

This retrospective analysis included all patients with a histologically confirmed diagnosis of PMBCL treated between January 2013 and December 2020 at two tertiary medical centers. Fifty‐six patients, incorporated in the study, were divided according to the frontline treatment regimen into dose‐dense R‐CHOP/R‐ICE and DA‐R‐EPOCH groups. The distribution of diagnosis years within the treatment groups was similar. The protocol was chosen according to discretion of the treating physician. The R‐CHOP/R‐ICE group received four cycles of R‐CHOP administered every 2 weeks followed by three cycles of R‐ICE.^28^ The DA‐EPOCH‐R group received a total of six cycles of this treatment.^24^ RT consolidation was given according to physician decision. Pre‐treatment clinical parameters, retrieved from the patient electronic medical records, included age, sex, date of diagnosis, constitutional symptoms, bulky disease, Ann Arbor stage, international prognostic index (IPI), the Eastern Cooperative Oncology Group (ECOG) performance status at diagnosis, presence of pleural/pericardial effusion, and extra‐nodal involvement. Institutional Review Boards of the participating centers approved the study protocols (approval number RMB‐D‐0209‐21). The primary endpoint of the study was PFS of PMBCL patients treated with frontline R‐CHOP/ R‐ICE or DA‐EPOCH‐R. Secondary outcomes included OS, end‐of‐treatment response (complete response (CR)/partial response (PR)/refractory disease), use of consolidative RT, and treatment‐related complications, including neutropenic fever and infections, as well as a total number of days of hospitalization due to treatment administration and acute toxicities subsequent to treatment. Response to therapy was assessed based on the end‐of‐treatment PET‐CT scan results using the Deauville score, with a complete metabolic response defined as Deauville score 1–3 and partial response, progressive disease, or refractory disease defined as Deauville score 4–5. The follow‐up policy for these patients involves periodic monitoring, including physical examination and blood tests (i.e., every 4 months in the first 2 years since completion of therapy and then every 6 months until 5 years). Imaging evaluation is not performed on a routine basis, and may be scheduled only at treating physician's request based on clinical grounds.

### 2.2 Statistical analysis

Categorical variables were presented with counts and percent and continuous variables were presented with median and interquartile range (IQR). Demographic characteristics of the study population and rates of adverse events were compared using the Fisher's exact test for dichotomous variables and the Wilcoxon rank‐sum test for continuous variables. Survival rates were estimated using the Kaplan–Meier analyses. OS was calculated from the date of diagnosis until the time of death or last follow‐up. PFS was calculated from the date of diagnosis until the time of progression, relapse, or last follow‐up. The median follow‐up was calculated from the date of diagnosis to the date of the most recent update (last follow‐up). Statistical analyses were performed using SAS 9.4 software (SAS Institute Inc.). A *p*‐value <0.05 was considered statistically significant.

## 3 RESULTS

### 3.1 Patient characteristics

A total of 56 newly diagnosed adult patients with PMBCL were identified in the institutional databases and included in the analysis. Median age was 32 years (range 18–62) and 68% were females. Patient characteristics are summarized in Table 1. Overall, 25 patients (44%) received the R‐CHOP/R‐ICE regimen and 31 patients (56%) were treated with the DA‐EPOCH‐R regimen. Prevalence of advanced stage disease (stages III–IV) was higher in the DA‐EPOCH‐R group (45%), compared to R‐CHOP/R‐ICE recipients (16%, *p* = 0.02), but the R‐IPI was balanced between the groups. Other baseline parameters were generally well‐matched between the two groups. The percentage of treatment completion was similar in both cohorts (84% and 81% in R‐CHOP/R‐ICE in DA‐EPOCH‐R groups, respectively).

**TABLE 1:** Patient characteristics at baseline

	R‐CHOP/R‐ICE	DA‐EPOCH‐R	*p*‐value
	*N* = 25	*N* = 31	
Median age (IQR), years	33.2 (30–41.8)	31.4 (26.4–36.7)	0.2429
Gender––female	17 (68%)	21 (67.74%)	0.9836
ECOG PS	0	0	0.4464
B symptoms	7 (28%)	13 (41.94%)	0.2793
Stage			0.0593
I	5 (20%)	7 (22.58%)	
II	16 (64%)	10 (32.26%)	
III	0 (0%)	2 (6.45%)	
IV	4 (16%)	12 (38.71%)	
Grouped stage III/IV	4 (16%)	14 (45.16%)	**0.0202**
Bulky disease (≥10 cm)	14 (56%)	19 (61%)	0.7871
Site of extension			0.3432
None	21 (84%)	20 (64.52%)	
Lung	3 (12%)	6 (19.35%)	
Sternum	1 (4%)	2 (6.45%)	
Lung and sternum	0 (0%)	3 (9.68%)	
Number of cycles			**<0.0001**
2–5	1 (4%)	4 (12.9%)	
6	3 (12%)	25 (80.65%)	
7+	21 (84%)	2 (6.45%)	
Extranodal site			
Bone	1 (4%)	6 (19.35%)	0.1165
Liver	1 (4%)	1 (3.23%)	>0.99
Lung	3 (12%)	9 (29.03%)	0.1225
Pancreas	2 (8%)	1 (3.23%)	0.5806
Kidney	2 (8%)	0 (0%)	0.1948
Adrenal	0 (0%)	0 (0%)	—
Any extranodal site	4 (16%)	10 (32.26%)	0.1625
BM involvement	0 (0%)	2 (6.45%)	0.4968
Pleural effusion			0.0871
None	9 (36%)	16 (51.61%)	
Pleural	1 (4%)	5 (16.13%)	
Pericardial	7 (28%)	2 (6.45%)	
Pleuro‐pericardial	8 (32%)	8 (25.81%)	
R‐IPI			0.3999
0	6 (24%)	5 (16.13%)	
1	14 (56%)	13 (41.94%)	
2	3 (12%)	7 (22.58%)	
3	2 (8%)	6 (19.35%)	
RT	5 (20%)	3 (9.68%)	0.4447

Abbreviations: BM, bone marrow; DA‐R‐EPOCH, dose‐adjusted rituximab, etoposide, prednisone, vincristine, cyclophosphamide, and doxorubicin; ECOG PS, ECOG performance status; IQR, interquartile range; R‐CHOP, rituximab, cyclophosphamide, doxorubicin, vincristine, and prednisone; R‐ICE, rituximab, ifosfamide, carboplatin, and etoposide; R‐IPI, revised international prognostic index; RT, radiotherapy.

### 3.2 Outcomes

**FIGURE 1:**
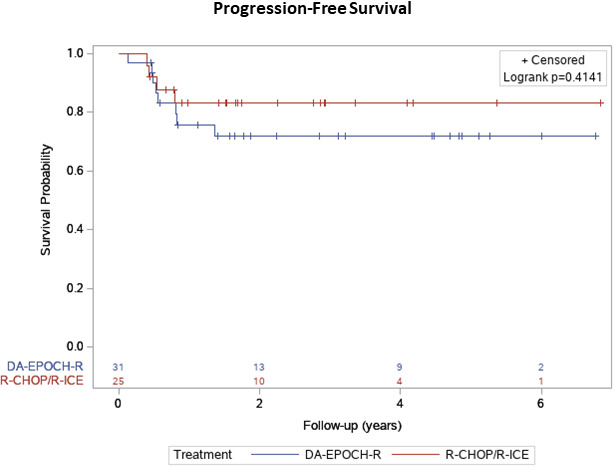
The Kaplan–Meier analysis of progression‐free survival depending on the treatment

**FIGURE 2:**
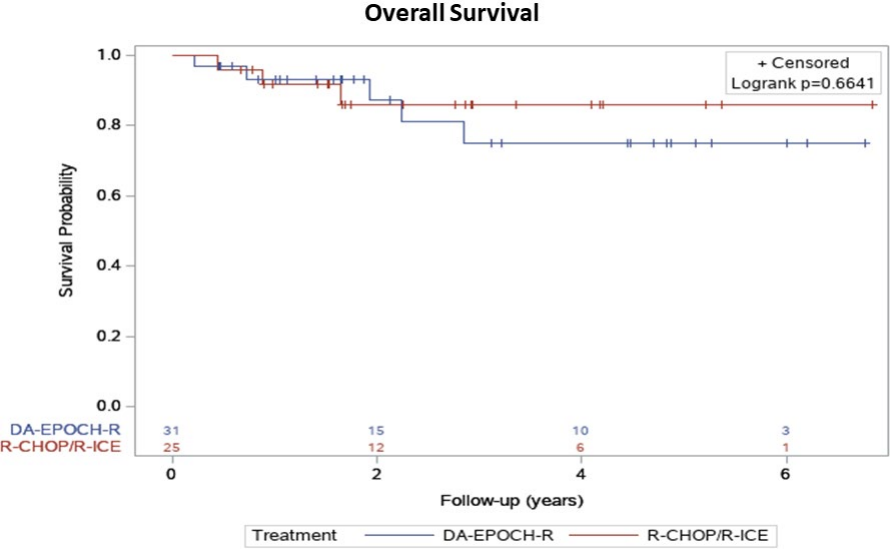
The Kaplan–Meier analysis of overall survival depending on the treatment

**FIGURE 3:**
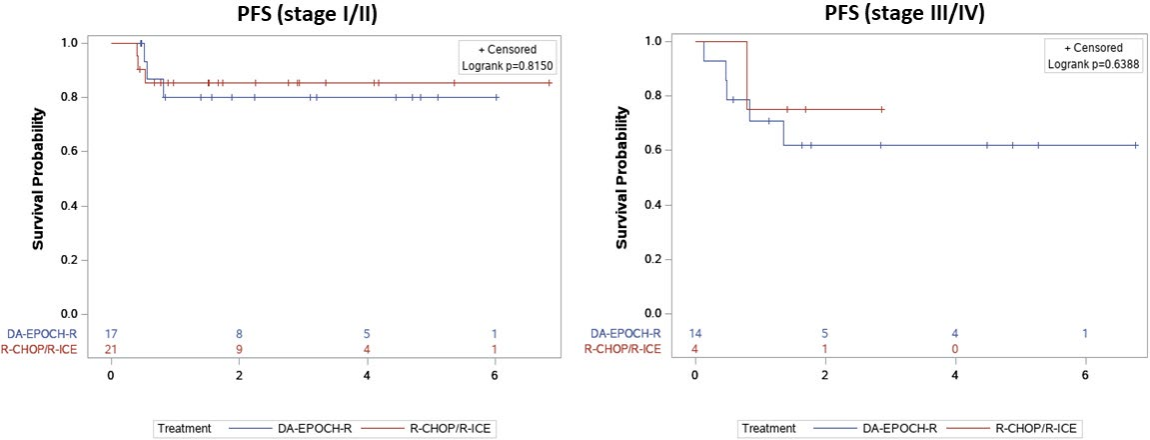
The Kaplan–Meier analysis of progression‐free survival depending on the treatment and stratified according to the disease stage (I–II vs. III–IV)

At a median follow‐up of 1.9 years (IQR 3.1, range 0.2–6.8 years), 22/25 patients (88%) in the R‐CHOP/R‐ICE group and 26/31 patients (83%) in the DA‐EPOCH‐R group are alive and disease‐free (Table 2). No significant difference in either PFS or OS was observed between the groups (Table 2), with a 2‐year PFS of 16% versus 26% (*p* = 0.37), median PFS of 1.7 years (IQR 0.8–2.9) versus 1.6 years (IQR 0.6–4.5; Table 2; Figure 1) and a median OS of 1.7 years (IQR 1.4–3.4) versus 1.9 years (IQR 1.1–4.7; Table 2; Figure 2) for recipients of R‐CHOP/R‐ICE and DA‐EPOCH‐R, respectively. As a significantly higher rate of an advanced stage disease was observed in the DA‐EPOCH‐R group, the Kaplan–Meier curve was stratified according to the cancer stage and again, no significant difference in PFS was found between the two study groups according to the log‐rank tests (Figure 3). The rate of refractory disease was similar in both groups, equating to 12% in the R‐CHOP/R‐ICE cohort and 12.9% in the DA‐EPOCH‐R cohort. Among the patients who received salvage therapy, three out of five individuals in the R‐CHOP/R‐ICE group and four out of six individuals in the DA‐EPOCH‐R group entered remission and were subsequently cured (Table 3). The proportion of patients who received additional RT after immunochemotherapy completion was similar in the two groups (20% in R‐CHOP/R‐ICE and 10% in DA‐EPOCH‐R group, *p* = 0.44; Table 1). Out of a total of eight patients who underwent RT, two patients from the R‐CHOP/R‐ICE group achieved CR and received RT as consolidation therapy (one for bulky disease and the other due to premature discontinuation of the R‐CHOP treatment related to the regimen toxicity). Among the remaining six patients, the indications for RT included refractory disease, partial response, or relapse.

**TABLE 2:** Study outcomes in the treatment groups

	R‐CHOP/R‐ICE	DA‐EPOCH‐R	*p*‐value
	*N* = 25	*N* = 31	
Death	3 (12%)	5 (16.13%)	0.7198
Progression and/or relapse	4 (16%)	8 (25.81%)	0.3740
Progression and/or relapse in 2 years	4 (16%)	8 (25.81%)	0.3740
PFS median (IQR), years	1.7 (0.8–2.9)	1.6 (0.6–4.5)	0.7667
OS median (IQR), years	1.7 (1.4–3.4)	1.9 (1.1–4.7)	0.8047
**Response to therapy**			>0.99
CR	20 (80%)	25 (80.65%)	
PR	2 (8%)	2 (6.45%)	
Refractory	3 (12%)	4 (12.9%)	
Mean no. of days in hospital for treatment administration	9.2 (3)	29 (7.1)	**<0.0001**
Mean no. of days in hospital due to treatment complication	5.4 (6.7)	5.5 (7.1)	0.8907

Abbreviations: CR, complete response; DA‐R‐EPOCH, dose‐adjusted rituximab, etoposide, prednisone, vincristine, cyclophosphamide, and doxorubicin; IQR, interquartile range; OS, overall survival; PFS, progression‐free survival; PR, partial response; R‐CHOP, rituximab, cyclophosphamide, doxorubicin, vincristine, and prednisone; R‐ICE, Rituximab, ifosfamide, carboplatin, and etoposide.

**TABLE 3:** Salvage treatments

Treatment	Age	Stage	R‐IPI	Response	RT	PFS (years)	Salvage therapy	Death	OS (years)
R‐CHOP/R‐ICE	30	IV	2	PR	Yes	2.9	ASCT	No	2.9
	18	II	1	Refractory	No	0.4	RGDP, Hyper‐CVAD	Yes	0.9
	32	II	1	Refractory	No	0.4	Bendamustine+ibrutininb, CAR‐T, ALLO SCT	No	4.2
	21	II	1	Refractory	Yes	0.5	DA‐EPOCH‐R x 1+ ASCT, nivolumab+ipilimumab	No	5.2
	30	IV	3	CR	No	0.8	ASCT	Yes	1.6
DA‐EPOCH‐R	35	II	0	Refractory	Yes	0.6	R‐GDP, CAR‐T cells, pembrolizumab	No	1.0
	29	III	2	Refractory	No	0.5	Bendamustin +brentuximab‐vedotin	Yes	0.7
	40	IV	2	CR	No	1.4	Pembrolizumab, CAR‐T cells	No	2.1
	34	II	1	PR	Yes	0.5	R‐ICE, ASCT	No	6.2
	19	IV	2	Refractory	No	0.5	R‐ICE, ASCT	Yes	1.9
	26	II	1	CR	No	0.8	R‐ICE, pembrolizumab+brentuximab‐vedotin, CAR‐T cells	No	1.7

Abbreviations: ART, radiotherapy; ASCT, autologous stem cell transplant; DA‐R‐EPOCH, dose‐adjusted rituximab, etoposide, prednisone, vincristine, cyclophosphamide, and doxorubicin; OS, overall survival; PFS, progression‐free survival; R‐CHOP, rituximab, cyclophosphamide, doxorubicin, vincristine, and prednisone; R‐GDP, rituximab, gemcitabine, dexamethasone, and cisplatin; R‐ICE, rituximab, ifosfamide, carboplatin, and etoposide; R‐ICE, rituximab, ifosfamide, carboplatin, and etoposide.

### 3.3 Toxicity

In the DA‐EPOCH‐R group, a median dose‐adjusted EPOCH level was 3 (range 1–6); in 32% of patients the dose was escalated to level 3, in 22% of patients the dose was escalated to level 4, and only 3 patients (10%) reached a dose‐adjusted EPOCH level above 4. Neutropenia with an absolute neutrophil count of <1000 cells/µl occurred in significantly more patients in the DA‐EPOCH‐R group than in the R‐CHOP/R‐ICE group, with the incidence of neutropenia equating to 97% and 64%, respectively (*p* = 0.003; Table 4). In terms of treatment‐related complications, the rates of infections, stomatitis, thrombotic complications, and febrile neutropenia were higher in the DA‐EPOCH‐R group, but the difference did not reach statistical significance (Table 4). Unlike the DA‐EPOCH‐R regimen, which requires inpatient administration, the R‐CHOP/R‐ICE regimen is administered in the outpatient setting. Hence, the use of R‐CHOP/R‐ICE has resulted in significantly reduced duration of hospitalization for treatment completion compared to DA‐EPOCH‐R (mean number of hospitalization days 9.2 [95% CI 8–10] vs. 29 [95% CI 27–31], respectively; [*p* < 0.001], Table 2). The mean number of hospitalization days due to treatment‐related complications was similar in the two groups.

**TABLE 4:** Adverse events in the treatment groups

	R‐CHOP/R‐ICE	DA‐EPOCH‐R	*p*‐value
	*N* = 25	*N* = 31	
**Adverse events**			
Infections	8 (32%)	14 (45.16%)	0.3161
Stomatitis	3 (12%)	9 (29.03%)	0.1225
Neuropathy	6 (24%)	8 (25.81%)	0.8767
Thrombosis	1 (4%)	4 (12.9%)	0.3670
Prolonged fever	1 (4%)	1 (3.23%)	>0.99
Elevated liver function tests	1 (4%)	0 (0%)	0.4464
Cardiotoxic	1 (4%)	0 (0%)	0.4464
Bowel obstruction	1 (4%)	0 (0%)	0.4464
Neutropenia	16 (64%)	30 (97%)	**0.0031**
Thrombocytopenia	12 (48%)	10 (32%)	0.2780
Hospitalization febrile neutropenia	11 (44%)	18 (58.06%)	0.2951
Hospitalization thrombosis	0 (0%)	1 (3.23%)	>0.99
Hospitalization chest pain	3 (12%)	4 (12.9%)	>0.99
Hospitalization stomatitis	0 (0%)	3 (9.68%)	0.2451
Hospitalization bowel obstruction	1 (4%)	0 (0%)	0.4464

## 4 DISCUSSION

PMBCL is a rare subtype of non‐Hodgkin lymphoma (NHL), occurring predominantly in the young female population. Despite the fact that PMBCL is considered an aggressive lymphoma, survival rates are significantly higher than in DLBCL and most patients are cured.^23^, ^24^, ^25^ Historically, treatment of PMBCL patients consisted of combined therapy using R‐CHOP + RT consolidation.^13^ However, given the high toxicity of RT, especially long‐term toxicity in the young adult population, it is imperative to identify the patients who could be cured with chemotherapy only without exposure to RT. Indeed, a recent prospective randomized trial conducted by the German Lymphoma Alliance,^21^ demonstrated a benefit of RT only in a subgroup of PMBCL patients who achieved PR, without any advantage in either PFS or OS for patients who achieved CR and received RT. Furthermore, the necessity and outcomes of additional radiotherapy as part of the standard treatment for PMBCL are being currently investigated in the ongoing international prospective randomized IELSG‐37 trial (https://clinicaltrials.gov/ct2/show/NCT01599559).

Both DA‐EPOCH‐R and sequential dose‐dense R‐CHOP/R‐ICE chemotherapy, previously assessed as an alternative regimen for administration of intensified chemotherapy, have been reported to be associated with highly promising clinical outcomes allowing to safely avoid additional RT in most PMBCL patients.^24^, ^27^, ^28^, ^29^ However, due to the disease rarity, data from prospective randomized controlled studies are scarce and the optimal therapeutic approach to PMBCL management is yet to be determined.

The current study has retrospectively compared the efficacy and toxicity of dose‐dense R‐CHOP/R‐ICE and DA‐EPOCH‐R chemotherapy regimens in newly diagnosed PMBCL patients. Overall, with a median follow‐up of 1.9 years, we observed excellent outcomes, in terms of PFS (2.1 vs. 2.4 years) and OS (88% vs. 83%), in both treatment groups. It is noteworthy that 80% of patients in each group achieved CR, and no intergroup difference in the frequency of refractory disease was observed. Importantly, these outcomes have been attained despite the low rates of RT utilization, that is, 20% and 10% of patients receiving R‐CHOP/R‐ICE and DA‐EPOCH‐R, respectively, in contrast to the reported 59% in patients treated with R‐CHOP.^25^ This provides another strong argument promoting broader application of the two former treatment modalities to clinical practice.

As for the toxicity profile of these regimens, our findings point to significant superiority of R‐CHOP/R‐ICE over DA‐EPOCH‐R in terms of neutropenia frequency (64% vs. 97%), with a trend for fewer cases of febrile neutropenia. R‐CHOP/R‐ICE is also associated with a trend toward lower rates of infections, stomatitis, and thrombotic complications. The observed differences in toxicity, are likely to be attributed, at least in part, to the mode of treatment administration. While DA‐EPOCH‐R is delivered as a continuous infusion, which requires hospitalization, R‐CHOP/R‐ICE may be administered in the outpatient setting. This has resulted in substantial reduction in hospitalization duration in the current study (9.2 vs. 29 days, respectively, *p*‐value < 0.001). Hence, the use the R‐CHOP/R‐ICE regimen in PMBCL patients might be associated with a lower treatment cost.

This analysis has several limitations, including its retrospective nature, a possible bias of treatment selection that was not randomized and was performed according to discretion of the physician, a small number of patients included in each group, and the relatively short median follow‐up period. However, despite the small size of the cohorts, patient baseline characteristics were similar in the two groups, aside from a higher proportion of patients with stage III–IV disease in the DA‐EPOCH‐R group (45% vs. 16%, *p* = 0.02). An extended follow‐up study, planned to be further conducted, is required to evaluate the long‐term toxicity profiles of both regimens, particularly those related to cardiotoxicity and secondary malignancies.

In conclusion, this study has demonstrated similar and highly promising clinical outcomes in terms of PFS, OS, and refractory disease frequency, in PMBCL patients treated with DA‐EPOCH‐R and R‐CHOP/R‐ICE. The R‐CHOP/R‐ICE regimen is found to provide lower rates of hematological and non‐hematological adverse events and a significant reduction in the hospitalization length. Considering the balance between toxicity and clinical efficiency, using of the R‐CHOP/R‐ICE regimen could be considered as a reasonable alternative to DA‐EPOCH‐R for the treatment of newly diagnosed PMBCL patients. Results of the ongoing randomized clinical trials may contribute to the establishment of an optimal PMBCL treatment strategy.

## CONFLICT OF INTEREST

The authors declare no potential conflict of interest.

## AUTHOR CONTRIBUTION

Y.M.: Designed and performed research, wrote the paper, and approved the final version of the paper. S.A.: Designed and performed research, wrote the paper, and approved the final version of the paper. N.G.: Performed research, interpreted the data, and approved the final version of the paper. ME. G. Interpreted the data, and approved the final version of the paper. B.N.: Designed research and approved the final version of the paper. N.A.H.: Designed and performed research. interpereted the data and approved the final version of the paper.

## ETHICS STATEMENT

This study was approved by the Institutional Review Boards (IRBs) of the participating centers (Approval #RMB‐D‐0209‐21) and was conducted in accordance with the 1964 Helsinki Declaration and its later amendments. The need for informed consent was waived by the IRBs due to the retrospective nature of the study.

## Data Availability

The data that support the findings of this study are available from the corresponding author upon reasonable request.
